# Does presbygeusia really exist? An updated narrative review

**DOI:** 10.1007/s40520-024-02739-1

**Published:** 2024-04-01

**Authors:** Valentina Ponzo, Mario Bo, Enrica Favaro, Fabio Merlo, Gianluca Isaia, Roberto Presta, Alessandro Collo, Sergio Riso, Simona Bo

**Affiliations:** 1 Department of Medical Sciences, University of Turin, Corso A. M. Dogliotti, 14, Turin, 10126 Italy; 2 Section of Geriatrics, AOU Città della Salute e della Scienza – Molinette, Turin, Italy; 3 Dietetic and Clinical Nutrition Unit, AOU Città della Salute e della Scienza – Molinette, Turin, Italy; 4 Dietetic and Clinical Nutrition Unit, AOU Maggiore della Carità, Novara, Italy

**Keywords:** Aging, Food choices, Food preferences, Presbygeusia, Taste alterations, Taste buds

## Abstract

This review critically assessed the existence of presbygeusia, i.e., the impairment in taste perception occurring in the elderly, as a natural part of the aging process and its potential clinical implications. Several factors might contribute to age-related taste alterations (TAs), including structural changes in taste buds, alterations in saliva composition, central nervous system changes, and oral microbiota dysbiosis. A comprehensive literature review was conducted to disentangle the effects of age from those of the several age-related diseases or conditions promoting TAs. Most of the included studies reported TAs in healthy elderly people, suggesting that presbygeusia is a relatively frequent condition associated with age-related changes in the absence of pathological conditions. However, the impact of TAs on dietary preferences and food choices among the elderly seems to be less relevant when compared to other factors, such as cultural, psychological, and social influences. In conclusion, presbygeusia exists even in the absence of comorbidities or drug side effects, but its impact on dietary choices in the elderly is likely modest.

**Supplementary Information:**

The online version contains supplementary material available at 10.1007/s40520-024-02739-1.

## Highlights

Taste alterations (TAs) – defined as abnormalities or changes in the sense of taste involving a modification in the perception of flavours, which can result in a diminished, altered, or distorted sense of taste – are common in older people.

TAs are probably a natural part of the aging process, so that the term presbygeusia may justifiably be used for defining abnormal taste perception of the elderly, in the absence of well recognized pathological or pharmacological conditions.

The clinical implications of TAs on health status and dietary choices of the elderly are likely modest, as many other factors have been reported to be ultimately more relevant in influencing food intake of old people.

**Supplementary Information:**

The online version contains supplementary material available at 10.1007/s40520-024-02739-1.

## Introduction

The term “presbygeusia” refers to the impairment of taste occurring in the elderly. Aging is a natural process affecting the entire body, resulting in a gradual decline in both physical and cognitive function [1]. The extent and rate of decline is highly variable among individuals, and include cognitive abilities, physical strength, cardiometabolic health, immune function, hormonal changes, and sensory functions [2]. Several sensory functions are affected by aging: vision (presbyopia), hearing (presbycusis), olfaction (presbyosmia) and taste.

Many age-related conditions might lead to impaired taste perception, such as changes in taste buds, decreased saliva production, alterations in the involved sensory nerves [3]. Although these changes occur physiologically during the aging process, impaired taste perception is not universally recognized as a natural phenomenon of aging, but rather as a pathological consequence of age-related chronic diseases (i.e., inflammation/infections in the oro/nasopharynx, post-traumatic or post-operative nerve damage, metabolic, endocrinological, neurologic and psychiatric diseases, malignancies, vitamin and mineral deficiencies, SARS-CoV-2 infection and burning mouth syndrome), environmental and chemical exposure, smoking habits, poor oral health, and polytherapy [3–15]. .

The main objective of the present narrative review was assessing the existence of presbygeusia as a distinct entity in the healthy elderly by an extensive literature search. Secondarily, the potential clinical implications of TAs in the elderly were discussed.

## Materials and methods

Studies investigating taste alterations in older adults (i.e., aged 65 years or older) without evident underlying pathological causes compared to healthy younger subjects were considered eligible for the present review. PubMed (National Library of Medicine), the Cochrane Library, Excerpta Medica dataBASE (EMBASE) and the Cumulative Index to Nursing and Allied Health Literature (CINAHL) were queried until 30 May 2023. The search strategy employed a combination of database-specific subject headings and keywords including (‘taste alteration*’ OR ‘taste dysfunction*’ OR ‘taste problem*’) AND (aging OR age-related OR elderly). No restrictions were applied during the search. Reviews, case reports, case series and conference proceedings as well as studies published in a language other than English and studies in animals were excluded but manually examined for additional relevant literature.

The identification of studies is summarized in Supplementary Fig. 1. The search strategy identified 837 studies; two investigators (SB and VP) independently screened titles and abstracts in duplicate for the selection of the included studies: 762 studies were excluded on title and abstract as clearly not relevant to the topic of the present paper. Then, the researchers independently assessed the full text of relevant studies and determined eligibility against the review topic. Only articles that explicitly assessed healthy individuals were included in the review. Any disagreement between the researchers in the study selection process was resolved through consensus or, if necessary, consultation with a third author (EF). A total of 15 observational studies were finally included in the review. The risk of bias was independently assessed for each included study by two authors (SB, VP) using the ROBINS-I (Risk Of Bias In Non-randomized Studies of Intervention) tool for observational studies [16].

Findings from the included studies were summarized in section “*Do taste alterations exist in healthy elderly?*”. All relevant studies, even those not meeting the eligibility criteria for the main objective of the review, were consulted to write background sections and discuss clinical implications.

### Definition of taste alterations

Taste alterations (TAs) refer to abnormalities or changes in the sense of taste involving a modification in the perception of flavours, which can result in a diminished, altered, or distorted sense of taste. Both quantitative and qualitative disorders of taste may be present [5]:

- Quantitative abnormalities.

Hypogeusia: diminished taste perception of one or more specific tastants (sweet, sour, salty, bitter, umami).

Ageusia: absent taste function.

Hypergeusia: increased sensitivity to taste stimuli.

- Qualitative abnormalities.

Dysgeusia: Altered perception of taste in response to a tastant stimulus.

Phantogeusia: perception of taste without a stimulus.

Aliageusia: taste disturbance in which a typically pleasant-tasting food or drink tastes unpleasant.

### Epidemiology

The prevalence of TAs widely varies with the population studied and the underlying cause. Moreover, it is strongly influenced by the diagnostic tool employed, i.e., subjective assessments (self-reported questionnaires or scales), taste testing (whole-mouth tests with solutions or spray or regional tests with taste strips) or electrogustometry [5].

Overall, these disturbances are relatively common, with percentages ranging from 5 to 20% in the general population, being complete ageusia relatively rare (< 3%) and men more frequently affected than women [5, 6, 17–22]. TAs are more commonly reported in older adults [17, 19, 23–26] with a prevalence between 10% and 30% [13, 27, 28]. Sour and bitter are the most compromised tastes in the elderly, while the perception of sweet is maintained [13, 24, 25, 28, 29]. A small study reported regional differences in taste perception in elderly adults, being the most affected areas both the tip and mid-lateral regions of the tongue (but not the posteromedial) [30]; conversely, another research found an increased age-related decline in taste function on the posterior tongue surface, especially for sweet and bitter tastes [31]. Overall, the right side of the tongue showed lower thresholds of taste perception than the left side [26]. These findings arise a further problem in the interpretation of the available literature, since regional tests might be unable to correctly detect TAs, depending on the site of application.

Additionally, individuals reporting TAs showed an associated impairment in the sense of olfaction, while an isolated loss of taste was described in less than 10% of patients requiring assistance for an impairment in flavour perception [4, 6]. Since the perception of a flavour is a complex sensorial experience, involving not only taste, but also tactile and chemical sensations, smell, and temperature perception [32], olfactory and tactile stimulations may be actually confused with taste, owing to the complexity in the peripheral and central regulation of the chemosensory inputs, which are combined into a unified flavour perception [6, 33].

### Pathophysiology

In addition to pathological causes and chronic diseases (which were not covered since they were beyond the purpose of the current review), several paraphysiological mechanisms have been proposed to explain the decreased taste sensitivity in older adults (Table 1).

**Table 1:** Mechanisms potentially implicated in presbygeusia

Factor	Implicated mechanism	Reference
Reduced fungiform papillae density	Reduced taste acuity perception	Fischer et al., 2013 [38] Pavlidis et al., 2013 [35] Piochi et al., 2018 [36] Piochi et al., 2019 [37] Walliczek-Dworschak et al., 2017 [35]
Reduced vascularization of the tip of the tongue and changes in shape of fungiform papillae	Reduced taste acuity perception	Pavlidis et al., 2013 [26]
Alteration of taste bud homeostasis; decreased number of taste buds, taste cells and taste progenitors/proliferating stem cells	Decline in taste sensitivity	Feng et al., 2014 [34]
Single nucleotide polymorphisms (SNPs) in the genes that code for the ENaC β subunit (SCNN1B gene) and the TRPV1 nonspecific cation channel (TRPV1 gene)	Modification in individual’s suprathreshold salt taste perception	Pavlidis et al., 2013 [26]
Decrease salivary flow rate and change in salivary composition	Reduced/altered taste perception	Walliczek-Dworschak et al., 2019 [35] Mese and Matsuo, 2007 [39]
Reduced salivary mucin glycosylation and binding capacity to oral epithelial cells	Impaired saliva viscoelasticity and decreased activation of bitter taste receptors	Pushpass et al., 2019 [40]
Specific alterations in brain areas and different neural signatures in the central nervous system	Reduced taste acuity perception	Iannilli et al., 2017 [41] Green et al., 2013 [42]
Reduced masticatory performance and number of missing teeth	Inability to fully chew food	Alia et al., 2021 [12]
Presence of dental prosthesis	Reduced ability to grind food and reduced rate of salivary secretion	Wayler et al., 1990 [43] Alia et al., 2021 [12]
Alterations in olfactory functions (drying of the olfactory mucosa, damage to the olfactory epithelium due to environmental factors, abnormal turnover with reduction in the number of olfactory receptor cells)	Abnormal retro-nasal stimulation of the olfactory receptors during deglutition with mitigation of the taste experience	Doty, 2018 [44]
Impairment in the oral microbioma	Impaired oral health Physical barrier exerted by surface tongue film of oral bacteria limiting access of tastants to taste receptors. Production of metabolites by oral bacteria Modification in saliva flow and pH	Belibasakis, 2018 [45] Schamarek et al., 2023 [46]

Structural changes have been reported with aging, such as a reduced number of taste buds, a lower epithelial density of taste buds, and fewer taste cells per taste bud [34]. Fungiform papillae (FP) on the anterior part of the tongue are the most studied papillae because of the accessible position and the association of their density with taste bud density and the perception of gustatory stimuli [26, 35, 36]. Several studies consistently demonstrated an age-associated decline in density of FP [26, 37, 38]. Furthermore, the function of FP may be impaired, since a reduced vascular density has been reported together with an altered vascular morphology at the tip of the tongue in subjects over 60 years old when compared with younger individuals [26]. Both impairment in taste bud homeostasis, i.e., abnormal cell renewal, differentiation, regeneration of damaged cells, and cell membrane modifications with dysfunction of taste receptors due to aging processes and the cumulative damage caused by harmful environmental conditions throughout life, might contribute to TAs [34].

Changes in the flow and composition of saliva, with impaired transport and release of food molecules to the taste buds have been reported in the elderly [39]. Reduced levels and glycosylation of salivary mucin together with a reduced binding capacity to oral epithelial cells were found in saliva of older adults with impairments in both saliva viscoelasticity and the activation of bitter taste receptors with respect to younger adults [40].

Brain electrical neuroimaging by means of scalp-recorded electroencephalography revealed specific alterations in brain areas implicated in the taste processes in healthy elderly people with respect to younger counterparts, suggesting the involvement of the central nervous system in aging-related TAs [41].

Functional magnetic resonance imaging (fMRI) activation during hedonic evaluation of sweet (sucrose) and bitter (caffeine) tastes were compared in 20 young (age 19–26 years) and 12 middle-aged (age 45–54 years) adults [42]. A greater bilateral activation in sensory (insula) and reward (lentiform nucleus) regions during evaluation of the sweet taste (but not bitter) was found in the younger group, thus suggesting that the early age-related decline in central processing of tastes may precede gustatory impairments of the elderly [42].

The inability to fully chew food [12], as well as the coverage of the palate with a dental prosthesis [43], might also be implicated in age-associated TAs.

Alterations in olfactory functions of the elderly, such as drying of the olfactory mucosa, damage to the olfactory epithelium due to environmental factors, abnormal turnover with reduction in the number of olfactory receptor cells, and the related abnormal retro-nasal stimulation of the olfactory receptors during deglutition might have an additional role in the impairment in taste perception [44].

Finally, impairment in the oral microbioma, which has been described in the older persons [45], has been increasingly recognized as a relevant factor in the mechanisms of altered taste perception by means of several mechanisms, such as the physical barrier exerted by surface tongue film of oral bacteria limiting access of tastants to taste receptors, the taste modulation by the bacterial metabolites, and the interaction of microbiota with extra-oral receptors [46].

### Do taste alterations exist in healthy elderly?

The purpose of our review was to investigate the current knowledge about the existence of presbygeusia as a distinct entity in the healthy elderly. A few studies explicitly assessed healthy individuals without comorbidities, conditions or drug therapies impacting on taste perception; in particular, drug use was often not considered among exclusion criteria. In 15 observational studies the most attention was paid to ruling out potentially interfering conditions with the sense of taste and were finally included in the review. Overall, the risk of bias was considered moderate for all included studies (Supplementary Table 1); a serious risk of bias was identified in three studies mainly due to the potential selection bias and the lack of control for confounding factors [5, 19, 31].

The main characteristics of included articles were outlined in Table 2, specifying the types of stimuli and the methods of taste assessment adopted. Twelve studies reported TAs in the healthy elderly people [5, 17, 19, 23, 26, 47–53], whereas three articles presented more controversial data [31, 43, 54].

**Table 2:** Evidence pros or cons the existence of presbygeusia

Evidence	Number	Age	Stimuli	Methods of taste assessment	Reference
**IN FAVOR**					
Patients with idiopathic taste disorders were significantly older than patients with post-traumatic taste disorders.	All: *n* = 491 256/235 M/F	9–92 y mean 56 y	Sucrose for sweet taste, sodium chloride for salty taste, quinine hydrochloride for bitter taste, citric acid for sour taste.	Whole-mouth test in 228 patients, test with taste strips	Fark et al., 2013 [5]
The recognition thresholds of all four basic tastes of elderly participants were significantly higher than those of young participants	OP: *n* = 30 12/18 M/F YP: *n* = 30 14/16 M/F	OP: 65–85 y YP: 18–29 y	Sucrose for sweet taste, sodium chloride for salty taste, tartaric acid for sour taste, quinine hydrochloride for bitter taste	Filter-paper disks soaked in each taste solution of all four basic tastes placed on the tip of the tongue	Fukunaga et al., 2005 [23]
A significant decline in thresholds was found in older for acetic acid, sucrose, citric acid, sodium and potassium chloride and inosine 5′-monophosphate (IMP)	OP: *n* = 21 10/11 M/F YP: *n* = 22 11/11 M/F	OP: 60–75 y YP: 19–33 y	Sodium chloride and potassium chloride for salty taste, sucrose and aspartame for sweet taste, acetic acid and citric acid for sour taste, caffeine and quinine hydrochloride for bitter taste, monosodium glutamate and inosine-5′-mono-phosphate for umami	Two-alternative forced-choice ascending detection method	Mojet et al., 2001 [47]
The absolute perception (intensity rating) decreased with age for all tastants in water, but only for the salty and sweet tastants in food products While wearing a nose clip, only the perception of salty tastants was diminished with age	OP: *n* = 21 10/11 M/F YP: *n* = 21 11/11 M/F	OP 60–75 y YP 19–33 y	Sodium chloride, potassium chloride, sucrose, aspartame, acetic acid, citric acid, caffeine, quinine, monosodium glutamate and inosine 5’-monophosphate dissolved in water and in ‘regular’ product (versions of commercially available iced tea, chocolate drink, mayonnaise, tomato soup and bouillon)	For each compound, the intensities of five ascending concentrations dissolved in water were compared with the intensity of the regular concentration of the compound in the product	Mojet et al., 2003 [48]
Increased electrogustometry thresholds from 60 years in the areas of the chorda tympani and glossopharyngeal nerves and from 70 years in the area of the greater petrosal nerve	OP: *n* = 158 70/88 M/F YP: *n* = 303 204/99 M/F	OP: 60–94 y YP: 15–60 y	Electrical stimuli	Electrogustometry	Nakazato et al., 2002 [17]
Higher electrogustometry thresholds at the tongue area innervated by the chorda tympani in the age group 60–69 years than in any of the younger age groups and, in older women, also at the soft palate area and at the area of the vallate papillae	OP: *n* = 30 15/15 M/F YP: *n* = 126 59/67 M/F	OP:>60 y YP: 10–59 y	Electrical stimuli	Electrogustometry	Pavlidis et al., 2013 [26]
The gustatory sensitivity decreases with age Age-specific taste loss was found for the taste qualities sweet and sour for both sides of the tongue, and bitter for the left side, which was more pronounced for sweet and sour	OP: *n* = 641 307/334 M/F YP: *n* = 303 139/164 M/F	OP: >56 y YP: 5–56 y	Sucrose for sweet taste, citric acid for sour taste, sodium chloride for salty taste, quinine hydrochloride for bitter taste	A drop (approximately 20 µL) of liquid tastant was applied on the right side or on the left side of the anterior/posterior third of the extended tongue	Pingel et al., 2010 [19]
Older adults perceived significantly lower taste intensities than young adults The perception of taste intensity increased slowly in the older group and remained lower than that of young adults	OP: *n* = 36 22/14 M/F YP: *n* = 45 22/23 M/F	OP: 60–81 y YP: 21–29 y	Sodium chloride solutions prepared with distilled water	Two sequential experiments: (1) a cup tasting test, in which participants sipped the solution from cups, spat it out, and rated with a 0–10 VAS static salty taste intensity (2) a time-intensity sensory evaluation, in which the solutions were delivered to participants’ tongues through a custom-made delivery system while they recorded dynamic taste intensities on a hand-held meter	Sato et al., 2022 [49]
Taste thresholds for amino acids were 2.5 times higher for elderly subjects than for young subjects	ns	OP: 75–87 y YP: 17–27 y	19 L-amino acids and four mono- hydrochloride derivatives dissolved in deionized water	Forced-choice ascending detection procedure	Schiffman et al., 1979 [50]
Thresholds for sweeteners were higher in the elderly when compared with the young	OP: *n* = 12 F YP: *n* = 12 F	OP 75–81 y YP 19–24 y	Acetosulfam, aspartame, cyclamate, fructose, neohesperidin, dihydrochalcone, rebaudioside, sodium saccharin, stevioside, thaumatin, D-tryptophan	Forced-choice ascending detection method	Schiffman et al., 1981 [51]
Both detection and recognition thresholds to a range of bitter compounds were increased in older subjects Significant losses in suprathreshold sensitivity to bitter tastants with age were also found	OP: *n* = 18 YP: *n* = 16	OP: mean age 81 y YP: mean age 27 y	caffeine, denatonium benzoate, magnesium chloride, magnesium nitrate, magnesium sulfate, naringin, phenylthiocarbamide, potassium nitrate, quinine hydrochloride, quinine sulfate, sucrose octaacetate, and urea	Forced-choice ascending detection method	Schiffman et al., 1994 [52]
Thresholds for all but sweet taste tended to gradually increase with age after the third decade.	OP: *n* = 114 56/58 M/F YP: *n* = 556 258/298 M/F	OP: 59–79 y YP: 10–59 y	Sucrose for sweet taste, sodium chloride for salty taste, tartaric acid for sour taste, quinine hydrochloride for bitter taste	The whole-mouth test	Yamauchi et al., 2002 [53]
**AGAINST or CONTROVERSIAL**					
No evidence that the perception of salt taste declines with age Older and younger subjects did not differ in their sensory evaluations of chicken broth, including ratings of the intensity of saltiness	OP: *n* = 24 12/12 M/F YP: *n* = 24 12/12 M/F	OP: 60–75 y YP: 20–30 y	Five salt solutions in water and eight samples of salted chicken broth containing from 0.04 to 0.64 mol/L sodium	A nine-point scale was used to rate the intensity and the acceptability of each solution presented in duplicate and in a random order	Drewnowski et al., 1996 [54]
The total correct quality identification scores of the whole mouth suprathreshold taste test and the quad taste test decreased with age, but the ability to identify sweet and salty tastes was not affected by age The age-related decline in taste function occurred mostly on the posterior tongue surface	All *n* = 240 OP: *n* = 80 40/40 M/F YP: *n* = 160 80/80 M/F	OP: ≥60 y YP: 20–59 y	Sucrose for sweet, citric acid for sour, sodium chloride for salty, caffeine for bitter, all dissolved in distilled water at different concentrations	Two solution-based taste tests were used: (1) the whole mouth suprathreshold test, and (2) the taste quad test, with application of a drop (15 µL) of the tastant solution onto one of the 4 quadrants of the tongue	Wang et al., 2020 [31]
Recognition thresholds for salt and sucrose were not significantly different between age groups Impaired suprathreshold functions for salt (but not for sucrose) were found in the older group	OP: *n* = 38 M YP: *n* = 37 M	OP: ≥65 y YP: <65 y	Aqueous solutions of sucrose and sodium chloride in deionized distilled water	Staircase method which incorporated a forced-choice paradigm to eliminate subject response bias	Wayler et al., 1990 [43]

Ns = not specified; OP = Older Participants; YP = Young Participants

In a small cohort of institutionalized older patients, selected by the nursing staff for their healthy medical status, the taste detection thresholds for amino acids [50], sweeteners [51], and bitter compounds dissolved in water [52] were reported to be higher than in younger subjects. Similar findings were observed with different methods of taste assessment [5, 19, 23], even if more controversial results were reported for saltiness [54] and sweetness [43]. In another small group of community-dwelling healthy non-smokers subjects, Mojet et al. found an age-related decline in the perception of intensity of all tastants dissolved in water, but only for the salty and sweet tastants in product [47]; when participants wore a nose clip to reduce the potential influence of the odour, an age-related decline in taste perception was found for salty tastants only [48]. However, in a different cohort the ability to identify sweet and salty qualities was reported to not be affected by age [34].

Furthermore, Nakazato et al. reported the increase of electrogustometry thresholds in older subjects at the chorda tympani and glossopharyngeal nerve areas from the age of 60 years, and at the greater petrosal nerve area after 70 years old [17]. Similar findings were reported by Pavlidis et al., with gustatory thresholds correlated with FP density, shape, and vascularization in older participants [26].

In summary, despite their inherent limitations, current evidence seems to lead towards the existence of presbygeusia as a relatively frequent condition related to the physiological decline of the body’s functions occurring with age.

### Clinical implications

TAs are unpleasant and disturbing conditions which may impact older people’s well-being by lowering their overall quality of life, decreasing their enjoyment of food, and impairing their ability to socialize when dining [6, 55]. Aging seems to result in a decline in taste function with tastant-dependent and not homogenous trend [29]. Physiological age-related decrease in gustatory function has been reported to be a slow and gradual process leading to a reduced awareness of TAs in individuals [13].

Age-related declines in taste may result in both a preference and consumption of stronger tasting products, as well as a loss of appetite, reduced food intake and undernutrition [3, 7]. However, the link between taste alterations, food preferences, and ultimately, food choices has not been proven, as neither preferences nor choices seem to be significantly influenced by TAs. Studies have indicated that older individuals, although experiencing some decline in sensory capabilities, exhibit a notable consistency in their food preferences, showing no preference for taste-enhanced food [54, 56]. A great variability of reported food preferences among the elderly with TAs has been reported, without clear-cut conclusions [57]. Food preferences may be more closely tied to culture or dietary customs rather than solely to sensory functions [58]. Moreover, the connections between food choices and intake with taste abnormalities were controversial, with many studies failing to establish a direct causal link [54, 59, 60]. Variations in taste receptor genes may result in different perceptions of taste and influence taste preferences. However, genetic predispositions only account for 20% of the variation in food preferences, with environmental factors playing a larger role [61]. Food preferences and choices are governed by a myriad of intricate processes, with sensory aspects (e.g., colour, smell, temperature, tactile and chemical sensations, in addition to taste) being involved alongside several other variables, such as mood, environment, health, allergies, convenience, hunger levels, cost, habits, cultural influences, social aspects, living conditions, attitudes towards food, religious beliefs, and life experiences [62]. Among the socio-cultural factors, income, education, country of origin, and knowledge of dietary/cooking skills were identified as the most significant influences of food choices in the elderly [63]. Other variables included biological, psychological, and situational factors (e.g., living alone, loss of a partner), as well as product characteristics (e.g., health claim, texture, price, portion size, promotions) [63]. Another systematic review highlighted influences from the past (e.g., childhood experiences and memories) and concerns about the future (e.g., loss of independence, fear of disability) as major drivers of food choices among the elderly [64].

### Limitations

The importance of measuring the intensities of tastants dissolved in water has been reported to have limited relevance for the actual perception of taste in complex food products in “real-life” situations [48]. Few studies have conducted repeated measurements of taste thresholds, which likely provide more reliable measures of actual sensitivity, revealing greater individual variability among the elderly [65]. However, several studies had methodological limitations and small sample sizes [25]. There has been a wide range of ages studied across different research, and older individuals have been found to be relatively inaccurate in self-reporting taste dysfunction [66]. The degree to which published results may be compared and generalized is uncertain, as considered studies have used various methods of taste assessment, different food systems, diverse procedures to collect responsiveness data (e.g., threshold or suprathreshold stimuli), and varying definition of abnormalities, both for detection thresholds (the minimum concentration at which participants can reliably discriminate the taste from water) and recognition thresholds (the minimum concentration at which participants can identify the taste quality) [67]. Finally, it is important to acknowledge the possibility of publication bias, where research with null findings is less likely to be published, potentially leading to an overestimation of the prevalence of TAs in the elderly.

## Conclusions

Our narrative review of the literature aimed to disentangle the effects of age from those of several age-related diseases or conditions potentially affecting taste. TAs should be regarded as a natural part of the aging process rather than a distinct disease, such that the term “presbygeusia” may reasonably be used to define abnormal taste perception of the elderly, in the absence of pathological conditions. However, the clinical relevance of TAs in the dietary choices of the elderly is likely to be limited, as many other factors eventually play a larger role in determining food intake in older adults.

### Electronic supplementary material

Below is the link to the electronic supplementary material.

Supplementary Material 1

## Data Availability

All Authors had all access to the data in this work and approved the submission of the present manuscript. All material in this assignment is Authors’ own work and does not involve plagiarism.
