# Supplementary material for: Does presbygeusia really exist? An updated narrative review
Source: Aging Clin Exp Res. 2024 Apr 1;36(1):84. doi: 10.1007/s40520-024-02739-1 (PMC10984891; doi:10.1007/s40520-024-02739-1)
Supplement: Supplementary file 1 — Supplementary Material 1 [file 40520_2024_2739_MOESM1_ESM.docx]

**
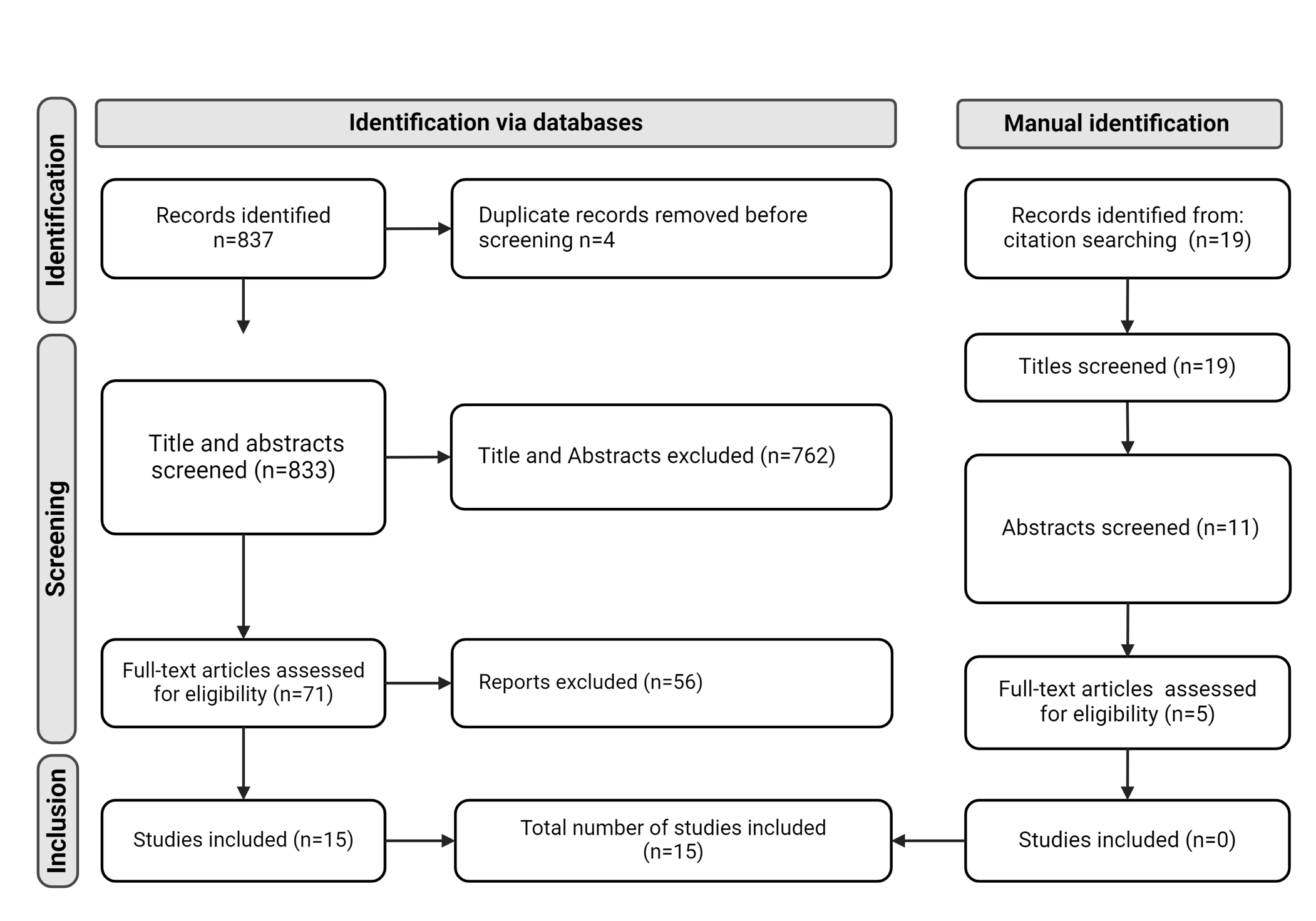
**

**Table S1. Risk of bias assessment of the observational studies according to ROBINS-I tool.**

| **Study** | **Bias due to confounding** | **Bias in selection of participants into the study** | **Bias in measurement classification of interventions** | **Bias due to deviations from intended interventions** | **Bias due to missing data** | **Bias in measurement of outcomes** | **Bias in selection of the reported result** | **Overall bias** |
| --- | --- | --- | --- | --- | --- | --- | --- | --- |
| Schiffman et al., 1979 | M | M | M | L | L | L | L | M |
| Schiffman et al., 1981. | M | M | M | L | L | L | L | M |
| Wayler et al. 1990 | M | M | L | L | L | L | L | M |
| Schiffman et al., 1994. | M | M | L | L | L | L | L | M |
| Drewnowski et al., 1996 | M | M | L | L | L | L | L | M |
| Mojet et a., 2001 | L | L | L | L | U | L | U | L |
| Nakazato et al., 2002 | M | M | L | L | L | L | L | M |
| Yamauchi et al., 2002 | M | M | M | L | L | L | L | M |
| Mojet et al., 2003 | L | L | L | L | U | L | U | L |
| Fukunaga et al., 2005 | L | L | M | U | L | L | L | M |
| Pingel et al., 2010 | S | S | M | L | U | L | U | S |
| Fark et al., 2013 | S | S | L | M | L | L | L | S |
| Pavlidis et al., 2013 | L | L | L | L | L | L | L | L |
| Wang et al., 2020 | S | S | M | L | L | L | L | S |
| Sato et al., 2022 | L | L | L | L | L | L | L | L |

L= Low risk of bias; M= Moderate risk of bias; S= Serious risk of bias; C= Critical risk of bias; U= No information
